# Intrinsically stretchable all-carbon-nanotube transistors with styrene–ethylene–butylene–styrene as gate dielectrics integrated by photolithography-based process

**DOI:** 10.1039/c9ra10534d

**Published:** 2020-02-25

**Authors:** Haoxuan Jiao, Min Zhang, Chunhui Du, Ziwei Zhang, Weihong Huang, Qiuyue Huang

**Affiliations:** School of Electronic and Computer Engineering, Peking University Shenzhen 518055 China zhangm@ece.pku.edu.cn

## Abstract

In recent years, stretchable electronics have attracted great attention because of their broad application prospects such as in the field of wearable electronics, skin-like electronics, medical transplantation and human–machine interaction. Intrinsically stretchable transistors have advantages in many aspects. However, integration of intrinsically stretchable layers to achieve stretchable transistors is still challenging. In this work, we combine the excellent electrical and mechanical properties of carbon nanotubes with excellent dielectric and mechanical properties of styrene–ethylene–butylene–styrene (SEBS) to realize intrinsically stretchable thin film transistors (TFTs). This is the first time that all the intrinsically stretchable components have been combined to realize multiple stretchable TFTs in a batch by photolithography-based process. In this process, a plasma resistant layer has been introduced to protect the SEBS dielectric from being damaged during the etching process so that the integration can be achieved. The highly stretchable transistors show a high carrier mobility of up to 10.45 cm^2^ V^−1^ s^−1^. The mobility maintains 2.01 cm^2^ V^−1^ s^−1^ even after the transistors are stretched by over 50% for more than 500 times. Moreover, the transistors have been scaled to channel length and width of 56 μm and 20 μm, respectively, which have a higher integration level. The stretchable transistors have light transmittance of up to 60% in the visible range. The proposed method provides an optional solution to large-scale integration for stretchable electronics.

## Abstract 2

In recent years, stretchable electronics have attracted great attention because of their broad application prospects such as in the field of wearable electronics, skin-like electronics, medical transplantation and human–machine interaction.

## Graphical abstract

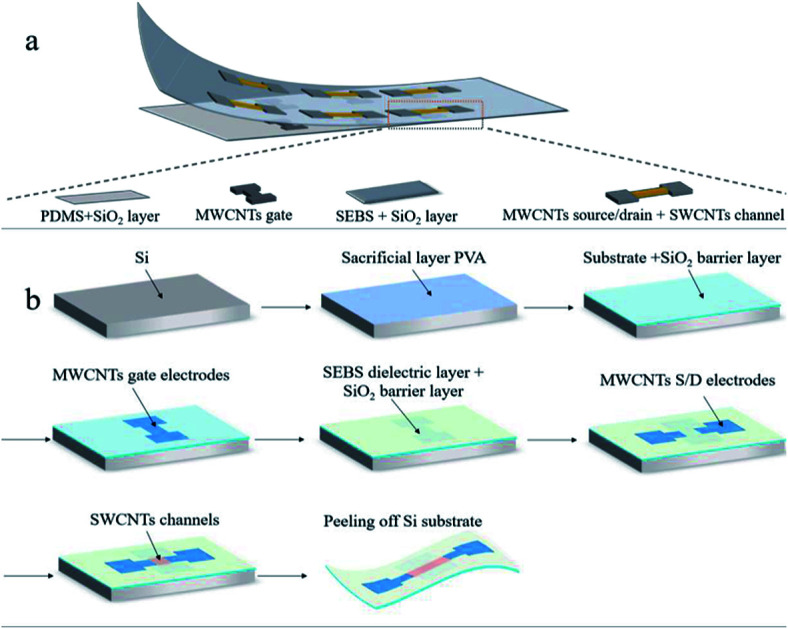

## 1. Introduction

Stretchable electronics have drawn lots of attention for their broad applications such as in wearable electronics, skin-like electronics,^1^ medical transplantation and human–machine interfaces. Stretchable thin-film transistors (TFTs) play an important role in rectification, switching, or amplification. Many remarkable works have made great contributions to the development of stretchable transistors. Haick *et al.* fabricated self-healing and multifunctional stretchable transistors with a relatively low operation voltage of 8 V.^2^ Fukuda *et al.* prepared highly stretchable transistors with high carrier mobility of 7.9 cm^2^ V^−1^ s^−1^ using solid-state elastomer electrolytes as the dielectric.^3^ Bao *et al.* fabricated intrinsically stretchable and scalable transistor arrays with the device density of 347 transistors per square centimeter.^1^ These efforts not only improved the performance of the stretchable transistors, but also proposed many novel processing methods to fabricate stretchable electronics such as printing, transferring, dip-coating *etc.* Nowadays, printing is becoming a promising method for preparing stretchable transistors because it allows large-scale and low-cost fabrication for electronic devices and circuits.^4^ However, most stretchable transistors fabricated by printing technology suffer from low carrier mobility, which greatly limits their application. Bao *et al.* have developed carbon-nanotube-channel stretchable transistors with high mobility up to 30 cm^2^ V^−1^ s^−1^, however, the channel width and length are 1 mm and 50–100 μm, respectively,^5^ which limits the capability of integration. As for other methods to fabricate stretchable transistors, transferring, dip-coating, or geometric engineering of non-stretchable components are difficult to realize high performance and small feature size simultaneously. Finding a way to fabricate transistors with high stretchability, high electrical performance, small feature size, and potential for mass production at the same time is vital to the development of stretchable transistors. To achieve this target, the traditional process based on photolithography and plasma etching has many advantages on process compatibility, equipment maturity, and low cost. However, it had been believed that intrinsically stretchable materials were not compatible with the traditional process because most organic materials are not resistant to plasma etching or ultraviolet light. That is why no work has been reported to fabricate stretchable electronic devices entirely by traditional photolithography–etching based process.

To address the problem mentioned above, in this work, we have introduced a plasma resistant layer to protect the polymer dielectrics and elastomer substrate from plasma etching or long-term UV light. Based on that, we have realized integratable stretchable all-carbon-nanotube thin film transistors by the traditional lithography–etching-based process platform. In this design, we adopt an organic material, styrene–ethylene–butylene–styrene (SEBS), as gate dielectric considering its lower viscosity and hysteresis performance,^1^ compared with polydimethylsiloxane (PDMS), poly urethane (PU) or other elastic materials. We use carbon nanotubes (CNTs) as channel and electrode materials in the all-carbon-nanotube transistors due to their excellent electrical properties with mean free path up to several micrometers and excellent mechanical flexibility.^6^ These all-carbon-nanotube transistors have been proved to obtain excellent electrical and mechanical performance. Zhang *et al.* fabricated ultralow-voltage flexible all-carbon transistors with carbon nanotubes as channel and electrodes, and graphene oxide as gate dielectric. These transistors showed high carrier mobility up to 105 cm^2^ V^−1^ s^−1^, extraordinary subthreshold swing of 170 mV dec^−1^, a low threshold voltage of −0.3 V, and a small bending radius of 1 mm.^7^ However, stretchable all-carbon transistors have not been reported before due to the obstacles during process integration. By addressing all the process obstacles, we have realized these high-performance integratable stretchable all-carbon-nanotube transistors. These transistors have shown excellent electrical and mechanical properties, with high carrier mobility up to 10.45 cm^2^ V^−1^ s^−1^, *I*_on/off_ current ratio more than 10^3^, and stretchability over 50%. More importantly, the transistors have a length and width of 20 μm and 56 μm, respectively, which have smaller area than most of the reported stretchable organic and CNT transistors, indicating its advantages for high-level integration.

## 2. Device realization and physical characterization

### 2.1 Fabrication of stretchable transistors

**Fig. 1:**
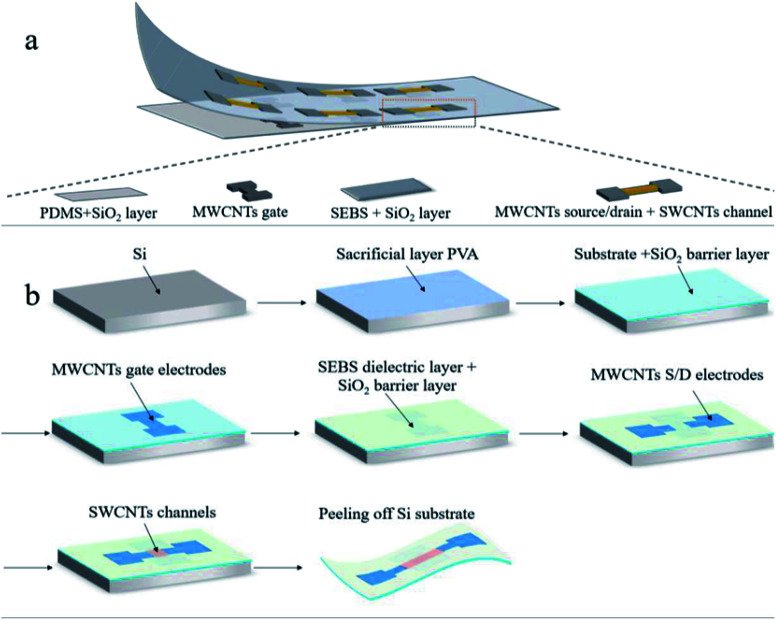
(a) The schematic diagram showing the components of the transistors. (b) Process flow for fabricating the intrinsically stretchable transistors based on photolithography and O_2_ plasma etching process.

Fig. 1a shows the schematic diagram of the proposed stretchable thin-film transistor. In this device, single-walled semiconducting carbon nanotubes (SWCNTs) (purity 99%) work as channel material and multi-walled metallic carbon nanotubes (MWCNTs) (0.92 wt%) work as source/drain/gate electrode materials. SEBS works as gate dielectric. PDMS works as the substrate for the stretchable transistors because of its high stretchability. There is a silicon dioxide (SiO_2_) layer deposited on SEBS dielectrics and PDMS substrate, respectively, working as the plasma-resistant layer. The schematic diagram of the transistor is shown in Fig. 1a. There are four main components, including PDMS substrate and SiO_2_ layer, MWCNTs gate, SEBS dielectrics and SiO_2_ layer, MWCNTS source/drain, and SWCNTs channel.

Fig. 1b shows the process flow of the transistors fabrication. Si wafer was utilized as the hard supporting substrate to facilitate the fabrication handling, and polyvinyl alcohol (PVA) (10 wt% water solution) was spin-coated on it at 2000 rpm, working as a sacrificial layer to facilitate the peeling off process later.^8^ On the PVA layer, we formed a PDMS layer of 700 μm thick by spin coating and a following curing process in an oven for two hours at 70 °C. After that, 50 nm SiO_2_ was grown on PDMS substrate at by PECVD at 150 °C to serve as O_2_ plasma-resistant layer. Then, the MWCNT gate electrodes of 20 nm thick were formed by spin-coating and defined by photolithography and O_2_ plasma etching. Next, the SEBS (60 mg ml^−1^ in toluene) was spin-coated at 3000 rpm on gate electrodes to form a 1.05 μm-thick dielectric layer. It is important for the SEBS dielectric layer to be placed in oven at 150 °C for 1 h to fully remove the water trapped in it, which is critical to reduce the gate leakage current. After that, the other 16 nm-thickness SiO_2_ used as O_2_ plasma resistant layer to protect the SEBS dielectric was deposited by PECVD at 150 °C. Then, 5 nm-thick MWCNT source/drain electrodes were formed using the similar process as that for gate electrode. After that, a SWCNT channel with a 16 nm thickness was formed by spin coating and defined by photolithography and O_2_ plasma etching process. Finally, we put the whole Si substrate into 60 °C water to remove PVA and peeled off the stretchable transistors membrane from the Si substrate. The SiO_2_ plasma-resistant layer could not be removed in this process.

### 2.2 Physical characterization of intrinsically stretchable transistors

**Fig. 2:**
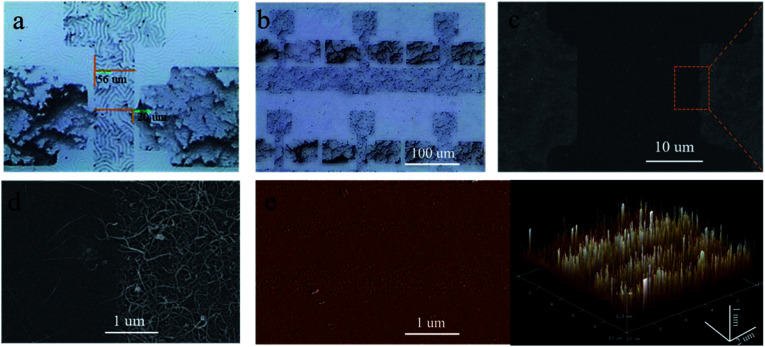
Physical characterizations of the transistors. (a) Optical microscope image of the intrinsically stretchable transistor in its initial state, with a channel length and width of 56 μm and 20 μm respectively. (b) Many transistors fabricated and integrated in a 9 cm^2^ substrate. (c) Top view SEM image of the transistor. (d) The magnified SEM view of the highlighted area. (e) 2D/3D AFM image of the SEBS/SiO_2_ layer, indicating that the roughness of the SEBS/SiO_2_ film is around 1.55 nm.

Fig. 2a is an optical microscope image of the intrinsically stretchable transistor in its initial state, and the transistor has a channel length and width of 56 μm and 20 μm, respectively. Source/drain electrode areas are relatively thicker metallic carbon nanotubes, although the surface is roughness, these can offer better conductivity. Fig. 2b shows many transistors fabricated and integrated in a 9 cm^2^ substrate. Fig. 2c is a SEM image showing the morphology of the as-fabricated transistor. Fig. 2d shows the magnified SEM view of the highlighted area in Fig. 2c. Distinction between the source/drain electrodes area and the channel area is clear. The sparse carbon nanotube network on the left is the channel area, and the relatively denser part of the carbon nanotube network on the right is the source/drain electrode area. Fig. 2e is the AFM characterization on the roughness of the SEBS/SiO_2_ film. Three dimensional (3D) structure shows that the average roughness of SEBS/SiO_2_ film is approximately 1.55 nm, which is super flat compared to the scale of the whole transistors and helps for improving the carrier mobility. There are periodical peaks and troughs due to the undissolved SEBS tiny particle.

## 3. Results and discussions

### 3.1 Electrical and mechanical characterization

**Fig. 3:**
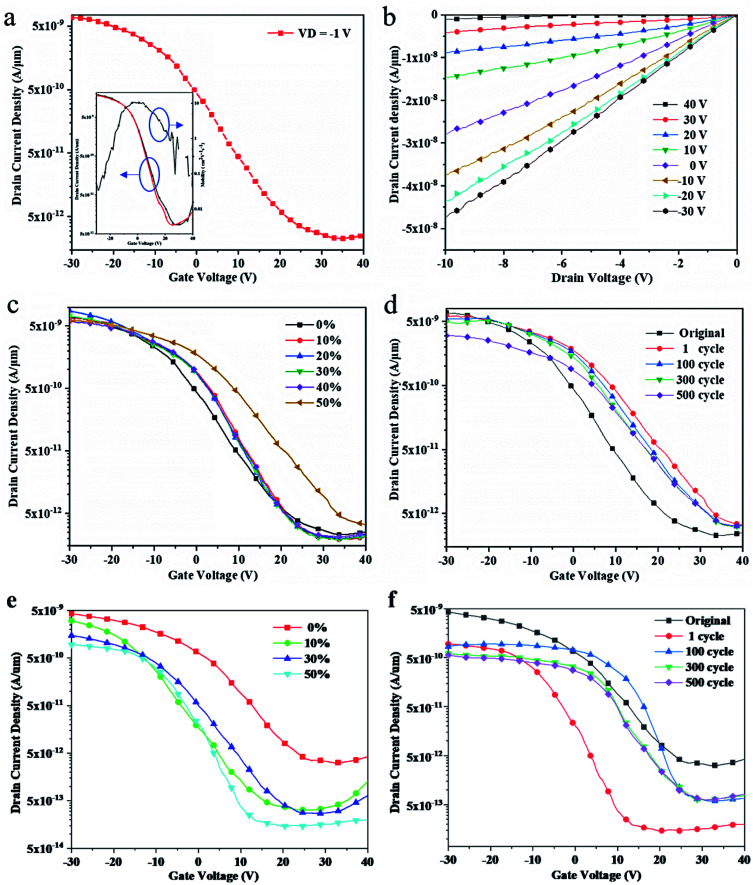
Electrical characteristics of the fabricated intrinsically transistors. (a) Transfer characteristics of the TFTs without strain. The inset includes the current hysteresis curves (left) and carrier mobility *versus* grid voltage (right) of the TFTs. (b) Output characteristics of the TFTs with *V*_G_ varying from −30 V to 40 V. (c) Transfer characteristics of the TFTs after various stretching along the channel length (*V*_D_ = −1 V). (d) Transfer characteristics of the TFTs after transistors are stretched along the channel length for various cycles at 50% strain (*V*_D_ = −1 V). (e) Transfer characteristics of the TFTs after various stretching perpendicular the channel length (*V*_D_ = −0.5 V). (f) Transfer characteristics of the TFTs, after stretching perpendicular the channel length for various cycles at 50% strain (*V*_D_ = −0.5 V).

Fig. 3a and b show the typical transfer characteristics and output characteristics of these transistors without applying strain, respectively. These transistors have only a little hysteresis, as shown in the inset of Fig. 3a. The carrier mobility *versus* grid voltage is also showed in the inset of Fig. 3a. Capacitance of the 1.05 μm-thick SEBS dielectric is 3.28 nF cm^−2^, and the calculated carrier mobility can reach up to 10.45 cm^2^ V^−1^ s^−1^. *I*_on/off_ is more than 10^3^. The working voltage of these transistors is from −5 V to −30 V (Fig. 3a). This is resulted from the low dielectric property of the SEBS.^9^ As far as we know, few works could achieve high carrier mobility, small size, and high stretchability at the same time. Chung and Bao *et al.* fabricated stretchable organic transistors with mobility 0.21–1.11 cm^2^ V^−1^ s^−1^.^10^ Hwang *et al.* fabricated stretchable transistors using PU as substrate and dielectric materials. These transistors could stretch to 160% but the mobility was just 0.034 cm^2^ V^−1^ s^−1^.^11^ Most importantly, the size of the transistors mentioned above are both large, which greatly limits their application in the field of integration.

Fig. 3c shows transfer characteristics at *V*_D_ of −1 V, after the transistors were stretched under different strains along the channel length. It is notable that the threshold voltage (*V*_th_) of transistors shifts slightly and the carrier mobility decreases to 6.92 cm^2^ V^−1^ s^−1^ under a 10% stretching strain. With transistors stretched under 20% strain, the *V*_th_ becomes −2.91 V and the carrier mobility drops to 6.43 cm^2^ V^−1^ s^−1^. The transistor performance changes slightly under 30% to 40% tensile strength, and the carrier mobility maintains at 5.71 cm^2^ V^−1^ s^−1^, while the *V*_th_ are −3.32 V and −1.66 V, respectively. When the transistor is stretched by 50%, the carrier mobility becomes 4.38 cm^2^ V^−1^ s^−1^ and the *V*_th_ becomes 1.5 V.

These TFT transistors can only be stretched by maximum 50% due to the process influence. The theoretical maximum stretchability of PDMS is 160% and the CNT network can be stretched by over 50%. However, the process to fabricate the stretchable transistors is harmful to the stretchability of PDMS substrate, including ultraviolet exposure, and high temperature disposal. Besides, the remaining SiO_2_ plasma-resistant layer also limits the stretchability of the devices. According to the Fig. 3d, the on-state current of the transistors decreases slightly and the threshold voltage shifts to the left under 50% tension for 300 times stretching. The carrier mobility decreases to 2.01 cm^2^ V^−1^ s^−1^ after stretching for 500 times. The data has shown that these transistors can still operate at a comparatively high mobility even after various stretching.

Fig. 3e shows the transfer characteristics of the transistor with channel length of 20 μm and width of 80 μm at different stretching along the direction perpendicular to the channel direction. We can see that the proposed transistor maintains certain stability after stretched under different tensions lower than 50%. Its carrier mobility can reach 3.34 cm^2^ V^−1^ s^−1^ compared with the initial mobility 8.78 cm^2^ V^−1^ s^−1^. The threshold voltage of the transistor shifts to the left after stretching. Fig. 3f shows the change of the transfer characteristics of this transistor under the stretching of 50% perpendicular to the channel at different stretching cycles. It can be found that the performance of the transistor is stable from the 100th stretching to the 500th stretching. The carrier mobility changes to 1.65 cm^2^ V^−1^ s^−1^ and the threshold voltage remains stable. The data show that the proposed transistor can also work and maintain stable performance after stretched along the direction perpendicular to the channel.

### 3.2 Transistors' statistical data

**Fig. 4:**
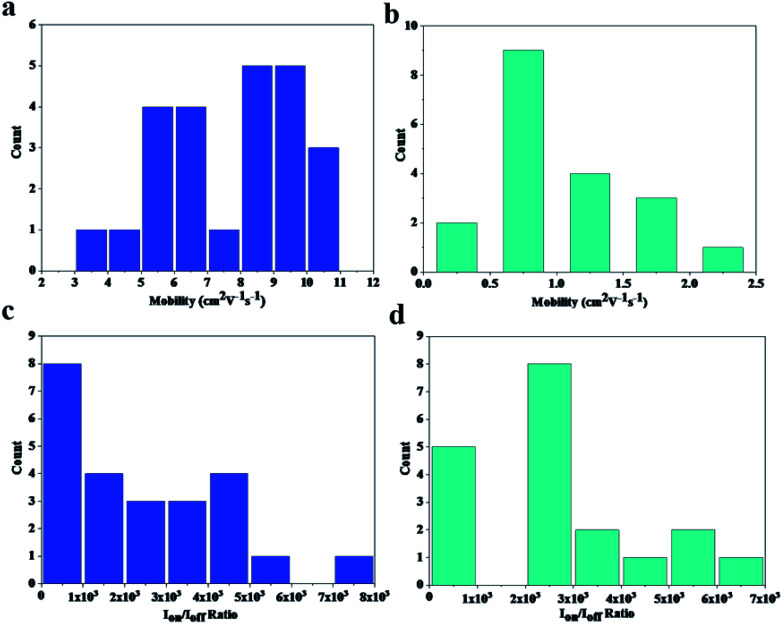
The statistical data of the proposed stretchable transistors. (a) The histogram for mobility of 24 stretchable transistors before stretching. (b) The histogram for mobility of 19 stretchable transistors after stretching by 500 cycles under 50% strain. (c) The histogram for *I*_on_/*I*_off_ of 24 stretchable transistors before stretching. (d) The histogram for *I*_on_/*I*_off_ of 19 stretchable transistors after stretching by 500 cycles under 50% strain.

We measured 24 transistors and there was 19 transistors can still work after stretching 500 cycles under 50% strain. Fig. 4a and b is the statistical data of mobility. We can see that after stretching most transistors' mobility can be over 1 cm^2^ V^−1^ s^−1^. The mean and standard deviation of the mobility before stretching is 7.65 cm^2^ V^−1^ s^−1^ and 2.03 respectively. After stretching 500 cycles, the mean and standard deviation of the mobility is 1 cm^2^ V^−1^ s^−1^ and 0.51 respectively. Fig. 4c and d is the statistical data of *I*_on_/*I*_off_ ratio. The mean and standard deviation of the *I*_on_/*I*_off_ ratio before stretching is 2526 and 1981 respectively. After stretching 500 times, the mean and standard deviation of the *I*_on_/*I*_off_ ratio is 2745 and 1750 respectively.

### 3.3 Transparency characterization

**Fig. 5:**
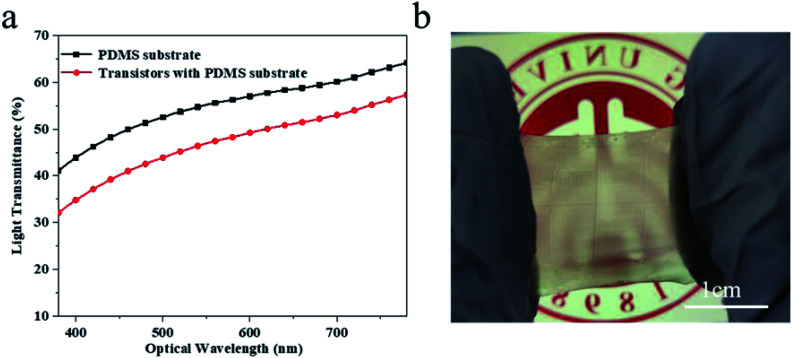
(a) The light transmittance of the transistors, reaching up to 60% in the visible range. (b) The intrinsically stretchable transistors, under a stretching of 50%.

The optical transparency of the device was also characterized. It can be seen from Fig. 5a that the light transmittance of the transistors membrane is approximately 55%, and the light transmittance of the transistors alone can reach 60%, indicating that the transistors have relatively high transparency. Fig. 5b is the transistors membrane in its state of stretching by 50%.

### 3.4 Comparison of intrinsically stretchable transistors

Table 1 compares this work with the state-of-art stretchable transistors. It is found that this work has achieved a good balance between carrier mobility, tensile property, and device feature size. These features make these transistors suitable for the certain applications simultaneously requiring speed, stretchability, and integration for stretchable electronics and circuits.

**Table 1:** Comparison of the intrinsically stretchable transistors fabricated in this work with the state-of-the-art works of stretchable transistors

Source/drain/gate/channel/dielectric materials	*W*/*L* (μm)	Stretchability (%)	Mobility before/after stretching (cm^2^ V^−1^ s^−1^)	Stretch cycles (times)	Ref.
Au sheet/Au sheet/Au sheet/P3HT fiber/ion gel	800/100	70	18/2.3	2000	12
MWCNTs/MWCNTs/MWCNTs/PTDPPTFT4 blend/SEBS	4000/200	100	1.32/0.2	100	9
MWCNTs/MWCNTs/MWCNTs/SWCNTs/SEBS	1000/50	80	0.12/0.06	400	13
MWCNTs/MWCNTs/MWCNTs/DPP-based polymer/PDMS	1000/50	100	1.27/0.1	1000	14
MWCNTs/MWCNTs/PEDOT:PSS/SWCNTs/PVDF-HFP	1000/50	20	30/14.5	1	5
MWCNTs/MWCNTs/MWCNTs/SWCNTs/ion gel	1000/8	100	13.5/5	1	15
Gold grid, PEDOT:PSS/gold grid, PEDOT:PSS/gold grid, PEDOT:PSS/SWCNTs/air dielectric layer	500/300	50	8.7/2.9	5000	16
AuNPs–AgNWs, PDMS/AuNPs–AgNWs, PDMS/AuNPs–AgNWs, PDMS/P3HT-NFs, PDMS/ion gel	1000/45–370	50	7.46 ± 1.37/3.57 ± 1.3	1	17
MWCNTs/MWCNTs/MWCNTs/C12-DDP/SEBS	2000/150	50	0.46/0.26	100	18
MWCNTs/MWCNTs/MWCNTs/P3HT, PDMS/CTBN	1000/100	34	0.61/0.08	1	19
Graphene/graphene/graphene/MnS_2_/Al_2_O_3_	40/10	4	0.56/0.32	1	20
MWCNTs/MWCNTs/MWCNTs/SEBS/SEBS	270/70	100	1.78/0.99	1000	1
AgNWs/AgNWs/PEDOT:PSS, PU/PVDF-HFP, PVP	2000/100	80	0.199/0.01	1000	21
MWCNTs/MWCNTs/MWCNTs/SWCNTs/SEBS	20/56	50	10.45/2.01	500	This work

## 4. Conclusions

In this paper, we have realized intrinsically stretchable transistors by traditional photolithography and O_2_ plasma etching based process. These transistors have small channel length of 20 μm and channel width of 56 μm, which is a prominent size for stretchable transistors. These transistors show a high carrier mobility of 10.45 cm^2^ V^−1^ s^−1^, with *I*_on_/*I*_off_ over 10^3^ on its initial state. Even after they are stretched by 50% tension for 500 times, they can still maintain relatively good electrical characteristics, with the mobility kept at 2.01 cm^2^ V^−1^ s^−1^ and the *I*_on_/*I*_off_ kept at 10^3^. This work provides a good solution to achieving high mobility, miniaturization and integration of stretchable transistors. Hopefully, this integration method based on the traditional process platform can play an important role in shaping the future of stretchable electronics.

## 5. Experiment part

The detail process of fabricating these stretchable transistors is showed in part 2.1 and the whole process is conducted in ultraclean room. The instrumentation used for electrical characterization is Agilent B1500A.

## Conflicts of interest

There are no conflicts to declare.

## Supplementary Material
